# Supplementary material for: 2-oxoglutarate triggers assembly of active dodecameric Methanosarcina mazei glutamine synthetase
Source: eLife. 2025 Mar 31;13:RP97484. doi: 10.7554/eLife.97484 (PMC11957540; doi:10.7554/eLife.97484)
Supplement: Figure 1—figure supplement 2—source data 1. [file elife-97484-fig1-figsupp2-data1.zip › Figure1-figure_supplement_2_source_data_labelled/Figure1-figure_supplement_2-source_data_labelled.pdf]

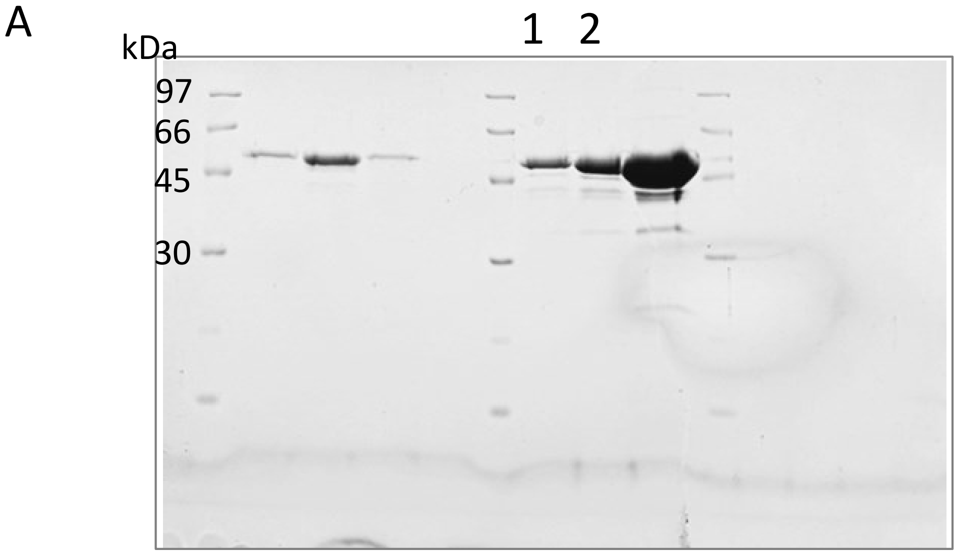

A: 1.5 µg (lane 1) and 3 µg (lane 2) Strep-GlnA1 on a coomassie-stained 12 % SDS-Gel.

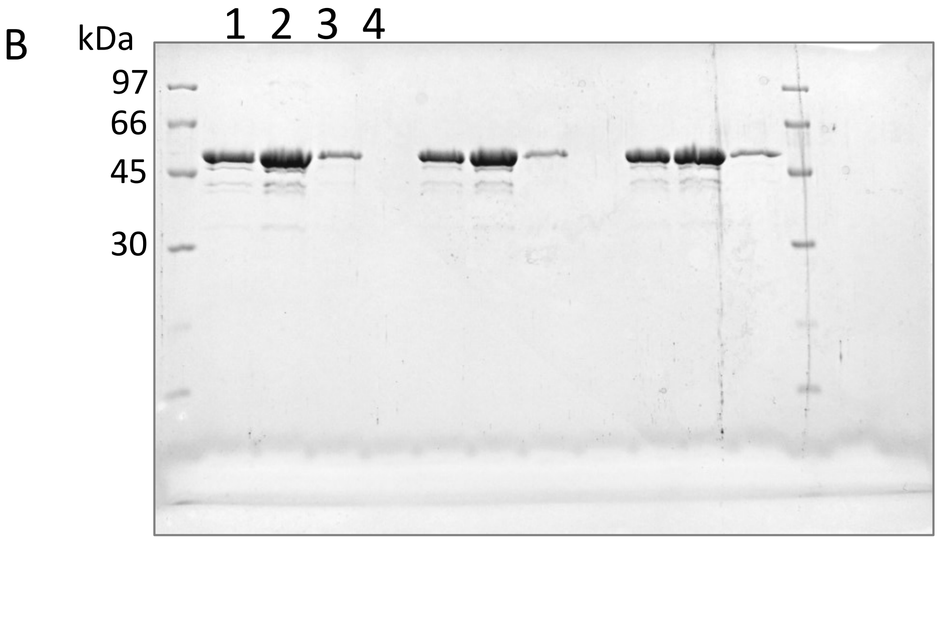

B: SEC-fractions of Strep-GlnA1 SEC-run; elution volume 1: 13.5-14 ml, 2: 14-15 ml, 3: 15-15.5 ml, 4: 19.5 – 20 ml on a coomassie-stained 12 % SDS-Gel
