# Supplementary figures and images for: 2-oxoglutarate triggers assembly of active dodecameric Methanosarcina mazei glutamine synthetase
Source: eLife. 2025 Mar 31;13:RP97484. doi: 10.7554/eLife.97484 (PMC11957540; doi:10.7554/eLife.97484)

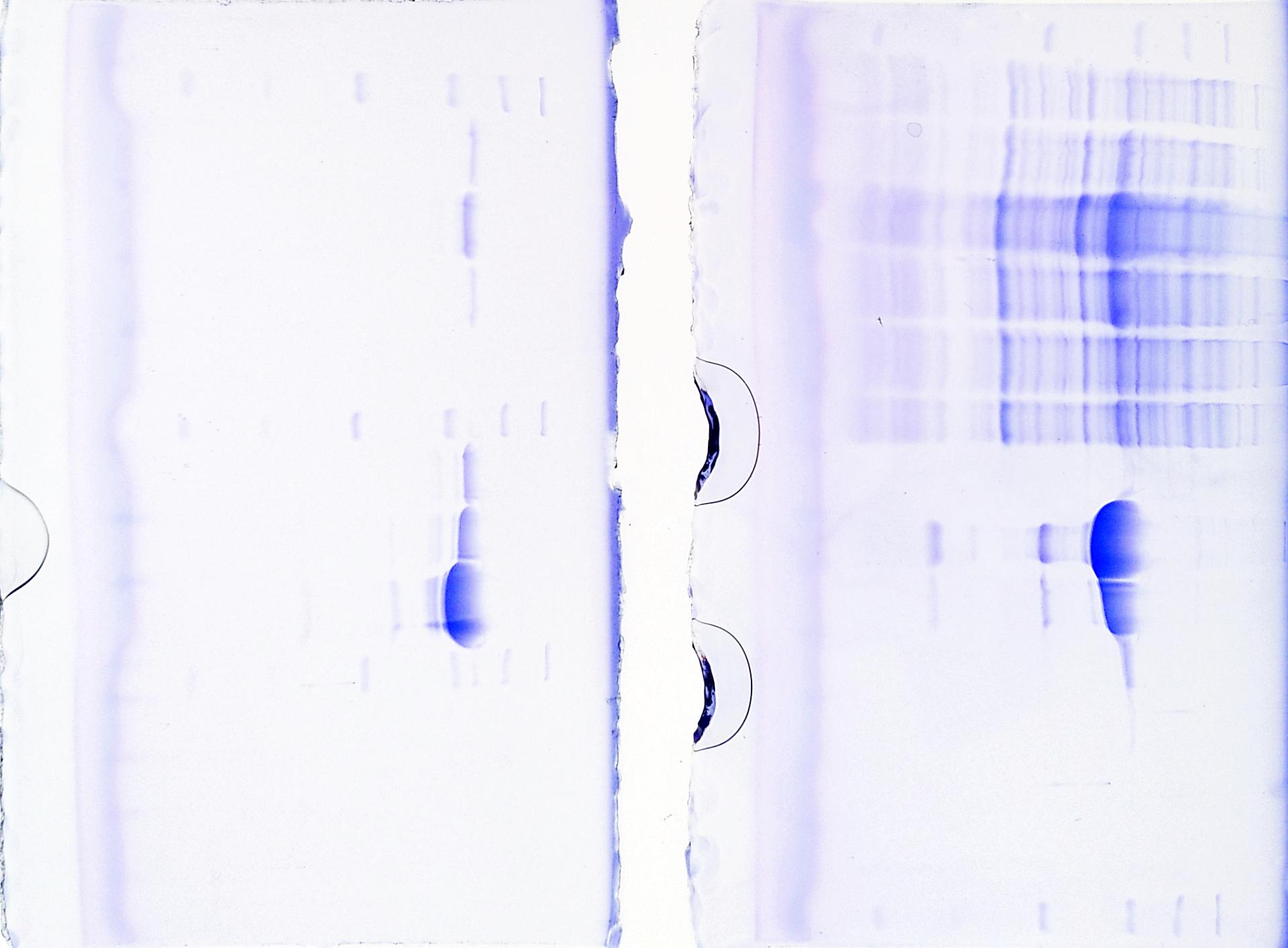

Supplement: Figure 1—figure supplement 2—source data 2. [file elife-97484-fig1-figsupp2-data2.zip › Figure1-figure_supplement_2_source_data_raw/Figure1-figure_supplement_2_source_data_1_raw.jpg]

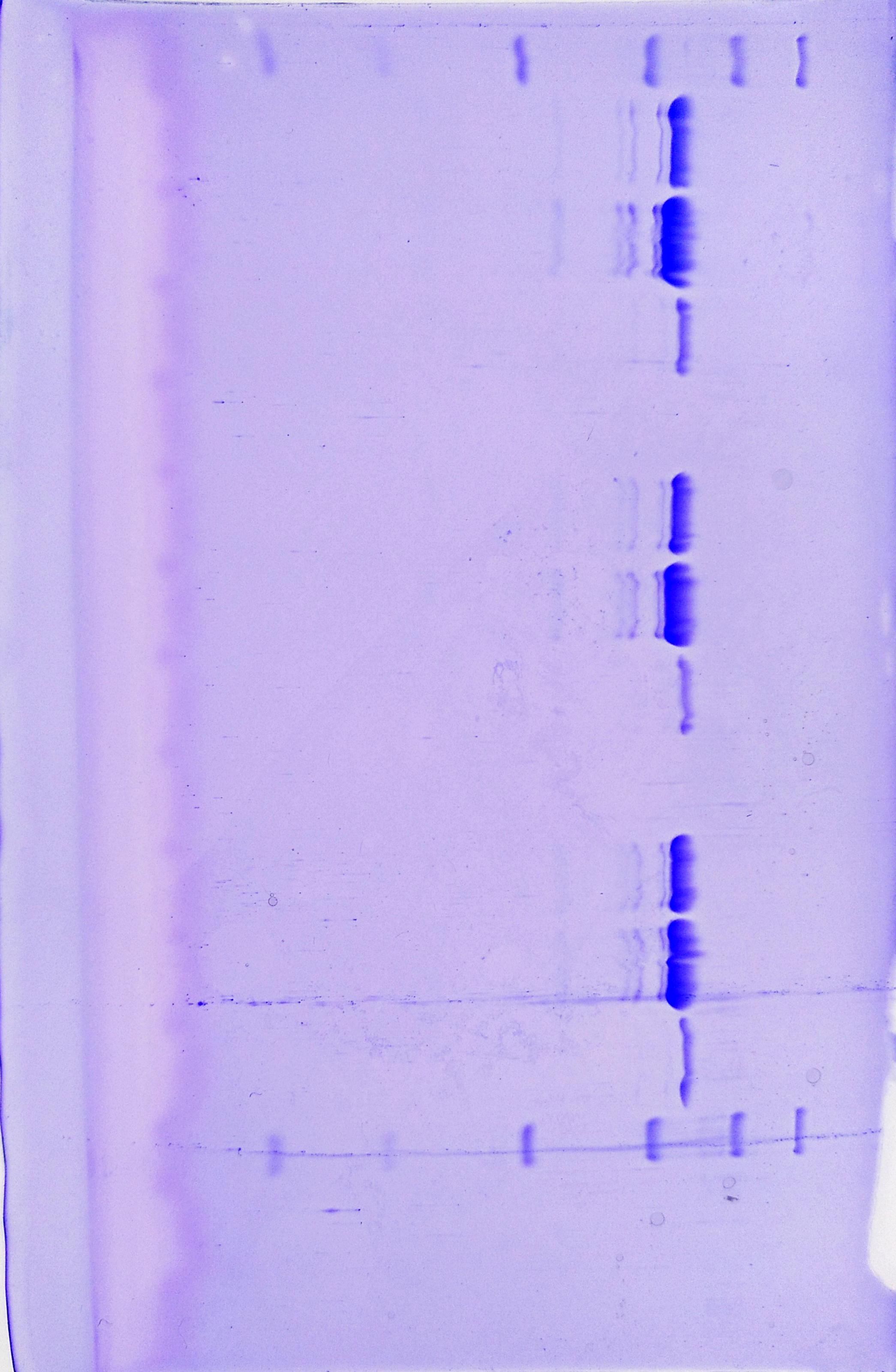

Supplement: Figure 1—figure supplement 2—source data 2. [file elife-97484-fig1-figsupp2-data2.zip › Figure1-figure_supplement_2_source_data_raw/Figure1-figure_supplement_2_source_data_2_raw.jpg]
