# Supplementary material for: 2-oxoglutarate triggers assembly of active dodecameric Methanosarcina mazei glutamine synthetase
Source: eLife. 2025 Mar 31;13:RP97484. doi: 10.7554/eLife.97484 (PMC11957540; doi:10.7554/eLife.97484)
Supplement: Supplementary file 1. [file elife-97484-supp1.docx]

Supplementary File 1: Strains and plasmids.

|  | **Properties** | **Reference** |
| --- | --- | --- |
| **Strains** | | |
| *E.* *coli* DH5α | General cloning strain | (Hanahan, 1983) |
| *E. coli BL21 (DE3)* | Strain for protein expression | Thermo Fisher Scientific, Waltham, USA |
| *E. coli BL21 (DE3)* + pRIL | Strain for protein expression of genes with unusual codons/ Cm^R^ | Stratagene, La Jolla, USA |
|  |  |  |
| **Plasmids** | | |
| pET21a | General cloning vector providing a C-terminal His_6_-tag | Novagen/Merck, Darmstadt, Germany |
| pETSUMO | Expression vector providing an N-terminal His_6_-SUMO-tag | Thermo Fisher Scientific, Waltham, USA |
| pRS375 | pET28a/Strep + GlnA_1_ | (Gutt et al., 2021) |
| pRS1672 | pETSUMO + GlnK_1_ | This work |
| pRS1728 | pEX-A258 + codon-optimized GlnA_1_ and sP26 | Eurofins Scientific, Ebersberg, Germany |
| pRS1841 | pRS375 + codon-optimized GlnA_1_ | This work |
| pRS1863 | pET21a + codon-optimized sP26 | This work |
| pRS1951 | pRS1840mut: R66AGlnA_1_ | This work |
