# Supplementary material for: 2-oxoglutarate triggers assembly of active dodecameric Methanosarcina mazei glutamine synthetase
Source: eLife. 2025 Mar 31;13:RP97484. doi: 10.7554/eLife.97484 (PMC11957540; doi:10.7554/eLife.97484)
Supplement: Supplementary file 2. [file elife-97484-supp2.docx]

Supplementary File 2: Cryo-EM data collection, refinement and validation statistics

|  | **Dodecamer Gln complex**  (EMDB- 19730)  (PDB 8S59) |
| --- | --- |
| **Data collection and processing** |  |
| Magnification | 165,000 x |
| Voltage (kV) | 300 |
| Electron exposure (e–/Å^2^) | 55.0 |
| Defocus range (μm) | 0.25-2.0 |
| Pixel size (Å) | 0.72 |
| Symmetry imposed | D6 |
| Initial particle images (no.) | 1,243,001 |
| Final particle images (no.) | 878,308 |
| Map resolution (Å)  FSC threshold | 2.39  0.143 |
| Map resolution range (Å) | 2.3-3.0 |
|  |  |
| **Refinement** |  |
| Initial model used (PDB code) | *de novo,* AlphaFold |
| Model resolution (Å)  FSC threshold | 2.5  0.5 |
| Model resolution range (Å) | 2.2-2.5 |
| Map sharpening *B* factor (Å^2^) | -80.1 |
| Model composition  Non-hydrogen atoms  Protein residues  Ligands | 46968  5352  AKG: 12 |
| *B* factors (Å^2^)  Protein  Ligand | 16.58  15.64 |
| R.m.s. deviations  Bond lengths (Å)  Bond angles (°) | 0.003  0.602 |
| Validation  MolProbity score  Clashscore  Poor rotamers (%) | 1.29  5.32  0.46 |
| Ramachandran plot  Favored (%)  Allowed (%)  Disallowed (%) | 98.22  1.56  0.23 |
